# Exploring protandry and pupal size selection for *Aedes albopictus* sex separation

**DOI:** 10.1186/s13071-018-3213-x

**Published:** 2018-12-24

**Authors:** Romeo Bellini, Arianna Puggioli, Fabrizio Balestrino, Marco Carrieri, Sandra Urbanelli

**Affiliations:** 1 Centro Agricoltura Ambiente “G.Nicoli” IAEA Collaborating Centre, Via Argini Nord 3351, 40014 Crevalcore, Italy; 2 Department of Environmental Biology, Sapienza University of Rome, Rome, Italy

**Keywords:** SIT, Sterile male, Genetic control, Mosquito, Dimorphism

## Abstract

**Background:**

We explored the possibility to improve male/female separation (sexing) in *Aedes albopictus* by selection of two strains, one toward increasing sex dimorphism and another toward increasing protandry. In the laboratory we selected and crossed small males with large females to exploit dimorphism, and early pupating males with late pupating females to exploit protandry.

**Results:**

While selection for enhanced dimorphism was not a profitable character, the selection for enhanced protandry up to F_10_ produced significant improvement in the time interval between male and female pupation. By collecting the pupae at 24 h from the beginning of pupation, without any sieving operation, we obtained about 28.50% of pupae (calculated in relation to the estimated initial number of first instar larvae used), *vs* 26.49% we had in the control strain, and, more interestingly, when checking the sex ratio of these pupae we observed a presence of females of 0.92% *vs* 23.02% in the control strain. We also modified our egg hatching protocol from the previous standard procedure that required keeping the eggs in the glass hatching container overnight (for about 14-16 h) to a new protocol where eggs are kept in the hatching container for 4 h in order to obtain more synchronized larvae. This was possible without any reduction in the egg hatching rate.

**Conclusions:**

In *Aedes albopictus* it is possible to develop hyper-protandric strains useful to produce male pupae without applying other sexing systems. This represents a considerable achievement assisting the Sterile Insect Technique application, allowing improvement of the current sexing method based on mechanical separation. More investigations are under way in order to further enhance the male productivity capacity of the strain and to determine whether the selection has any impact on the male fitness parameters.

## Background

Because of its vectorial capacity for several arboviruses like dengue, chikungunya and Zika [1, 2], *Aedes albopictus* is causing major public health concern in many regions with different climatic condition, from tropical to temperate areas. The surprising adaptation capacities of *Ae. albopictus*, from the original East-Asia rainy forest to nowadays metropolitan environments, exploiting a huge variety of small water collection, is indicative of the genetic background resources of this species [3, 4].

Despite significant efforts to develop vector control strategies well suited to local socio-economic conditions, currently available methods appear weak against urban mosquitoes, showing an unsatisfactory level of population reduction [5]. The situation is even more complicated in Europe, where the EU Biocide regulation N. 528/2012 [6] is progressively pushing insecticides out of the market, with two main effects: (i) weakening the vector control capacity because of the restricted availability of effective and long lasting active ingredients; and (ii) increasing the possibility of raising resistance because it becomes more and more difficult to rotate insecticides with different mechanism of action [7, 8].

Alternative or complementary to insecticides, mosquito control methods such as the genetic-based control strategies targeting the reproductive capacity of vector mosquitoes are under investigation, strongly supported by the new DNA based knowledge and tools, offering a spectrum of possible applications [9, 10].

Among the genetic control methods, the Sterile Insect Technique (SIT), despite being only known from about 60 years, is currently considered a classic genetic control method, having achieved considerable successes against many dangerous insect species in the last decades [11].

The SIT strategy requires only the release of males, which are made functionally sterile in order to induce sterility in the target female population. The release of only males is particularly important in the species where the female is the responsible of harmful to the agricultural production or to the human and animal health, as in the case of mosquitoes. An efficient sexing system is therefore required allowing the separation between sexes assuring high rates of male recovery with very low or absence of any residual females [12].

This has been achieved through many different systems depending on the specific features of the species, targeting genetic, mechanical, behavioural and more recently molecular components. Reviews of the methods proposed in the past to sex mosquitoes have been published by Papathanos et al. [13] and Gilles et al. [12].

During the SIT application programme started in Italy in the year 2000 targeting *Aedes albopictus* [14], a mechanical sexing system exploiting size dimorphism at the pupal stage in the water has been developed and utilized for pilot trials [15]. Unfortunately, this system is far from being satisfactory as it allows to recover 22-30% only of the total available males with a residual presence of females of 0.5-1.0%. The low male recovery rate strongly impacts the production cost while the residual presence of females is not acceptable especially in endemic countries where they may contribute to disease transmission. We therefore started to look for better performing sexing methods and conducting laboratory studies focusing on the possibility to enhance two naturally occurring phenomena that are known to be useful for sex separation: pupa size dimorphism and protandry which is the faster development of males.

Sexual dimorphism is a widespread trait occurring in many animals and some plants. In insects the most common sexual dimorphisms pertain to size, ornamentation and coloration [16].

By definition protandry is the emergence of adult males before females of the same species. It was first described by Darwin [17] as a trait under sexual selection that favours mating probability of males while reducing the pre-reproductive time of females [18, 19]. It is a widespread phenomenon both in animals and plants [20].

Models indicate that there is stabilizing selection on male emergence time because late-emerging males miss opportunities to mate and males that emerge too early risk death before they have the opportunity to mate [21, 22]. Bradshaw et al. [23], working on *Wyeomyia smithii* came to the conclusion that at least in this species, protandry is a heritable feature capable of responding to selection.

Following Wiklund & Fagerström [24], protandry is a reproductive strategy of males mainly occurring in species where the female is monogamous. A review of protandry in nature is proposed by Morbey & Ydenberg [20].

To our knowledge, increasing protandry has never been considered as a possible feature exploitable in sexing insects for SIT purposes.

## Methods

### Selection for dimorphism

An *Ae. albopictus* strain originated from eggs collected in the field in Rimini Province (northern Italy) was used for this study (strain Rimini F40). The rearing was carried out in a climatic room at a temperature of 28 ± 1°C, 80% RH and 14:10 (L:D) photoperiod. The larvae were reared in white plastic trays (30 × 10 × 40 cm) at a density of 4000 larvae in three litres of water and were fed for four days from first instar larvae with a diet consisting of 80% Friskies cat food, 14% Tetramin fish food and 6% brewer’s yeast (CAA standard diet). For more details on rearing please refer to Balestrino et al. [25]. At each generation, at approximately 24 h and 48 h from the beginning of pupation, pupae in the water were sieved two times: once at 24 h by using a metal sieve with 1250 μ mesh in order to obtain small male pupae only (pupae which passed through the sieving mesh), and then at 48 h by using another metal sieve with 1550 μm mesh in order to obtain large female pupae only (collected among the pupae which did not pass through the sieving mesh). From generation F_2_ the metal sieves were changed to 1180 and 1700 μm to separate male and female pupae respectively. Each generation, 250 small male pupae and 250 large female pupae were collected randomly, checked individually under a stereomicroscope to avoid any possible presence of pupae of undesired sex and placed into a cage (40 × 40 × 40 cm) for mating and egg production. The pupae of intermediate size were discarded. Pupa production and the percentage of females present after sex separation (conducted at 24 h after pupation onset using a 1400 μm sieve) were observed on the strain before the selection for dimorphism (control strain) and after 3 generations of selection. Another mechanical sieving was conducted on the dimorphic strain using a 1180 μm sieve.

### Egg hatching procedure

During preliminary studies, we realized that the hatching protocol in place, which required the eggs being left overnight in a glass jar with nutrient broth [25], may affect detection of protandry. We therefore conducted a study to evaluate the possibility of reducing the hatching duration by leaving the nutrient broth solution overnight in sealed glass jars without the eggs, introducing the eggs the next morning and checking the eclosion rate at 1, 2, 3 and 4 h. Two *Ae. albopictus* strains were used for this study, both strains were maintained in laboratory at standard conditions (28 ± 1°C, 80% RH and 14:10 L:D photoperiod), one originated from eggs collected in the field in Rimini province (northern Italy) and was reared for 67 generations (Rimini F_67_), while the other strain originated from eggs collected in the field in Montenegro and was used for the experiment after one generation in the laboratory (MNE F_1_) (courtesy of Igor Pajovic, University of Montenegro). The hatching rate obtained at each time interval was compared with the hatching rate obtained with our standard procedure (control). Three replicates for each treatment were performed.

### Selection for protandry

An *Ae. albopictus* strain originated from eggs collected in the field in Forlì-Cesena province (northern Italy) was used for this study (strain Cesena F_1_). The rearing was carried out in a climatic room at a temperature of 28 ± 1°C, 80% RH and 14:10 (L:D) photoperiod. The eggs were hatched for 4 h in jars containing deionized water together with the hatching solution (nutrient broth and brewer’s yeast), which were left closed overnight before introduction of the eggs. The larvae were reared in white plastic trays (30 × 10× 40 cm) at a density of 2 larvae/ml (4000 larvae in two litres of water) and were fed for four days from L1 with a diet consisting of 50% tuna meal, 36% bovine liver powder, 14% brewer’s yeast and 0.2% w/v of Vitamin Mix (Vanderzant Vitamin Mix, Bio-Serv, Frenchtown, NJ) (IAEA-BY diet) [26]. At each generation, 250 first pupating pupae (males) and 250 last pupating pupae (females) were collected, sexed under the stereomicroscope and placed into a cage (40 × 40 × 40 cm) for mating and egg production. The intermediate pupating pupae were discarded. No mechanical separation was therefore applied during the selection process.

At generation F_3_ and F_10_, we checked the pupal production (number of pupae produced in relation to the initial number of reared L_1_) and the percentage of females present, before and after mechanical sieving, at 24 h after pupation onset. We also checked the sex ratio of pupae collected at approximately 24, 48 and 72 h to investigate the male and female production of the strain (percentage of males and females produced in relation to the initial number of L_1_). The data were compared with the data produced by the same strain before selection and with a control strain maintained in standard laboratory condition for 67 generations (Rimini F_67_). Three replicates were performed for each treatment.

In order to evaluate the size of the males before and after the sex separation procedure, the wings of males from the hyper-protandric strain (HYPRO) and the control were measured. The right wing (or left if the right was damaged or lost) was removed under a dissecting microscope from a sample of individuals from each strain. Each wing was measured from the distal edge of the alula to the end of the radius vein excluding fringe scales [27]. A digital image of the wing was made using a camera (uEye, iDS Imaging Development Systems GmbH, Obersulm, Germany), mounted on a stereomicroscope, and lengths were measured with ImageJ software (ImageJ, U.S. National Institutes of Health, Bethesda, MD).

### Statistical analyses

The pupal production and the percentage of contaminant residual females after the sieving procedure were compared between the control and the dimorphic strain, using different mesh size, by one-way analysis of variance (ANOVA).

The mean percentages of hatched eggs were compared between the standard protocol (control) and shorter time intervals (1, 2, 3 and 4 h) for Rimini F_67_ and Montenegro F_1_ mosquito strains. Data were analysed by two-way ANOVA.

The percentages of male and female production (calculated on L1 initial number) were compared between Rimini F_67_ (control) and the enhanced protandric strain (HYPRO F_10_) at different time intervals (24, 48 and 72 h) by ANOVA.

Likewise, the efficiency of mechanical sex separation was analysed by comparing both the pupal production of control and HYPRO F_10_ strains and the percentage of contaminant residual females before and after the sieving procedure.

The percentages were transformed for the analysis using an arcsine transformation. Means were separated by Newman-Keuls multiple comparison test. Male wing size of control and HYPRO F_10_ strains, before and after sieving, were compared using t-test for independent samples.

## Results

### Selection for dimorphism

The dimorphism selection protocol provided no evidence of possible linkage of the dimorphism on the sex genetics. After three generations of selection, we obtained a strain with both male and female pupae of smaller size in comparison with the initial strain. In fact, the sex separation conducted on the dimorphic strain using a sieve of 1400 μm mesh allowed a pupal production of 30.93%, much higher than the pupal production obtained with the control strain, before the selection for dimorphism (11.81%, standard error, SE 0.93), but with a residual female presence significantly higher compared to the control (*F*_(2, 3151)_ = 64.70, *P* < 0.001) (Table 1). Sieving again the pupae of the dimorphic strain with a sieve of 1180 μm mesh, resulted in a pupal production and a residual presence of females not significantly different to F_0_.

**Table 1:** Main pupal production parameter obtained with the selection for dimorphism

Generation	No. of pupae processed	Mesh size (μm)	% Pupal production on L_1_ (± SD)	% Females (± SD)
F_0_	1468	1400	11.81 (±2.07)^a^	1.29 (±11.31)^a^
F_3_	1237	1400	30.93 (±46.22)^b^	9.22 (±28.94)^b^
F_3_	449	1180	11.23 (±31.57)^a^	0.22 (±4.72)^a^

Abbreviations: L_1_, first-instar larvae; SD, standard deviation

Within a column, different letters indicate statistically significant difference (*P* < 0.001)

### Egg hatching procedure

**Fig. 1:**
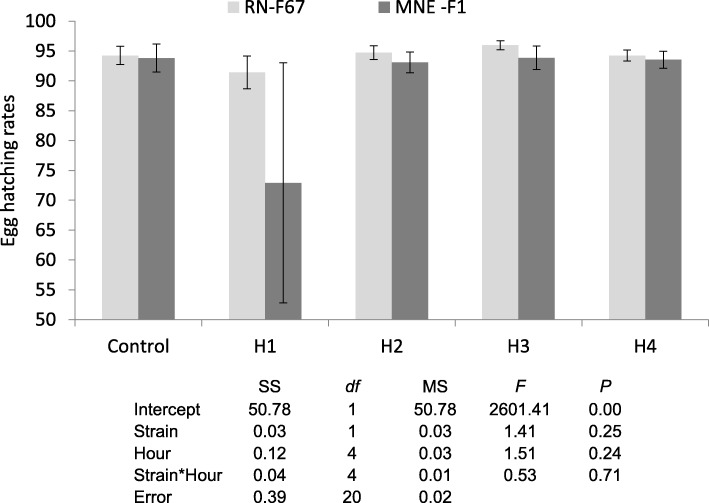
Egg hatching rates in two *Aedes albopictus* strains tested at different egg hatching time

The analysis performed by two way ANOVA showed no significant differences (Current effect: *F*_(4, 20)_ = 0.534, *P* = 0.712) in the hatching rate obtained by applying the standard protocol (control) or shorter hatching time treatments, in both strains tested (*P* < 0.05), but higher variability were observed at 1 h hatching time in comparison with the longer hatching times tested. The data are shown in Fig. 1.

### Selection for protandry

The selection conducted for an increased interval between male and female pupation time showed that the male and female production in the pupae collected at 24, 48 and 72 h from the pupation onset, in generation F_10_ of the hyper-protandric HYPRO strain, is significantly shifted in favour of males compared to the initial colony used as the control (Table 2). In F_10_, collecting pupae at 24 h from the beginning of pupation, without applying any sex separation procedure, we obtained a pupal production of 28.53 ± SE 2.85% (calculated as the number of pupae produced in relation to the number of estimated starting L1 larvae) against 26.49 ± SE 1.6% we had in the control strain. More importantly, when checking the sex ratio of these early pupating pupae we saw that in HYPRO F_10_ we had a presence of females of 0.92 ± SE 0.1%, against 23.02 ± SE 0.78% in the control. After the sex separation procedure, the pupal production was 11.77 ± SE 0.52% with no residual females for the HYPRO strain, against 9.43 ± SE 0.8% with 0.61 ± SE 0.48% residual female in the control strain (Table 3).

**Table 2:** Pupation dynamics evaluated at three time intervals for two *Aedes albopictus* strains

		% Male production on L_1_			% Female production on L_1_		
Strain	N	24 h (±SD)	48 h (±SD)	72 h (±SD)	24 h (±SD)	48 h (±SD)	72 h (±SD)
Control	5	20.24 (±1.02)^a^	13.29 (±1.25)^a^	1.9 (±0.06)^a^	6.26 (±0.60)^a^	16.82 (±0.97)^a^	6.18 (±0.41)^a^
Hypro F_10_	3	28.28 (±2.84)^b^	9.37 (±1.38)^a^	1.49 (±0.39)^a^	0.26 (±0.02)^b^	5.40 (±0.62)^b^	8.84 (±1.42)^a^

Abbreviations: L_1_, first-instar larvae; SD, standard deviation; Control, laboratory reared strain Rimini F_67_; Hypro F_10_, protandric strain with data collected at generation F_10_;

Within a column, different letters indicate statistically significant difference (*P* < 0.05)

**Table 3:** Pupal production and residual presence of females, at 24 h from pupation onset, in two *Aedes albopictus* strains, before and after sex separation

		Before sieving		After sieving	
Strain	N	% pupal production on L_1_ (±SD)	% female (±SD)	% pupal production on L_1_ (±SD)	% female (±SD)
Control	5	26.49 (±1.60)^a^	23.02 (±0.78)^a^	9.43 (±0.80)^a^	0.61 (±0.48)^a^
Hypro F_10_	3	28.53 (±2.85)^a^	0.92 (±0.10)^b^	11.77 (±0.52)^a^	0.00 (±0.00)^a^

Abbreviations: L_1_, first-instar larvae; SD, standard deviation; Control, laboratory reared strain Rimini F_67_; Hypro F_10_, protandric strain with data collected at generation F_10_;

Within a column, different letters indicate statistically significant difference (*P* < 0.05)

The male wing length was compared, by checking before and after mechanical sieving, finding no evidence of any difference between the HYPRO and the control strains (Table 4).

**Table 4:** Wing length of *Aedes albopictus* males belonging to two different strains, obtained from pupae collected at 24 h from the pupation onset, measured before and after mechanical sieving with 1400 μm

		Male wing size (μm)		
Strain	N	Before sieving (±SD)	N	After sieving (±SD)
Control	109	2202 (±6)^a^	89	2167 (±8)^b^
Hypro F_10_	144	2256 (±6)^a^	147	2235 (±5)^b^

Abbreviations: SD, standard deviation; Control, laboratory reared strain Rimini F_67_; Hypro F_10_, protandric strain with data collected at generation F_10_;

Within a line, different letters indicate statistically significant difference (*P* < 0.001) using t-test for independent samples

## Discussion

Separation of sex in order to be able to release male only, remains an important critical issue to be solved on the way to apply SIT in mosquito suppression. In this study we explored the possibility to exploit two features which are well known to be present in many mosquito species: sexual dimorphism and protandry. While we were not able to select a strain of *Ae. albopictus* with increased size difference between male and female, thus possibly allowing better separation between sexes, it appears that protandry has some useful advantages.

Protandry, defined as the earlier sexual maturation of males compared to con-specific females, is a common phenomenon in many animal taxa including fish, amphibians, reptiles, birds, mammals and insects. The adaptive significance of protandry is not fully understood and several explaining hypotheses have been proposed [20]. To better clarify the adaptive significance of sex-biased development time, it might be necessary to better analyse the costs-benefits for the males to be ready for mating earlier than other males and than females. The most relevant factors that seems to play a selective role on the protandry are considered the degree of multiple mating by males and the occurrence of male territoriality [20]. In *Ae. albopictus*, it is known that male can mate several times while female has only a small rate of polygamy [28, 29]. No information is available about male territoriality in mosquitoes.

Protandry is naturally present in *Ae. albopictus* and commonly observed in the laboratory where rearing condition can be standardized and therefore early pupating individuals are usually males.

The idea we pursued was to select a strain with an increased dimorphism at the pupal stage and another strain with an increased time window separation between males and females. Both these characters may allow for a better sex separation in mass rearing facilities serving the production of sterile males. While the dimorphism character seems not responding to the selective pressure as conducted, the selection toward enhanced protandry provided highly encouraging results.

The data we obtained indicate the possibility to develop a hyper-protandric (HYPRO) *Ae. albopictus* strain allowing high male recovery rate coupled with very low residual presence of females. With the F_10_ HYPRO strain and without any sieving sex separation operation, we recovered about 57% of the reared males with a residual presence of females of less than 1%. The data obtained at F_10_ are considerably better when compared with the current sieving method which allow the recovery of 22-30% of the reared males with a residual presence of females in the range 0.5-1.0%.

The HYPRO strain is potentially able to allow the sex separation without mechanical sorting but will require a method to separate pupae from larvae and a mass rearing scheme able to maintain the trait under mass rearing condition.

Another advantage possibly related with the exploitation of hyper-protandric strains in SIT programmes, could be that because it is not necessary to apply any separation dealing with sex dimorphism, it might be possible to release males in a larger size range. As indicated by the male wing size comparative analysis we have performed on initial strain and the HYPRO F_10_ strain, the selection for protandry does not have influence on the male size.

More investigations are going on to understand the possible impact of this selection procedure on the general fitness of the strain and on the performance of the produced males. The association of HYPRO strain with *Wolbachia* transinfection deserves further exploration in the incompatible insect technique perspective.

During the study we also tested the possibility to reduce the time required for egg hatching, from the previous overnight protocol to a much shorter protocol of 1, 2, 3 and 4 h. Results indicated that very good hatching rates can be obtained even at shorter hatching time, which might result to be less stressful for the newly hatched larvae.

## Conclusions

The selection for an *Ae. albopictus* hyper-protandric strain with characters highly useful for sex separation in mass rearing facility serving SIT programmes, may open a new way improving the cost-benefit ratio of genetic control. This easily applicable method may support better productivity in mass rearing facility while allowing the release of males without any size selection. The protandry, being a trait largely present in many mosquito species may find application in the mass production of other mosquito species currently under SIT development.
